# Development of a Sandwiched Piezoelectric Accelerometer for Low-Frequency and Wide-Band Seismic Exploration

**DOI:** 10.3390/s23229168

**Published:** 2023-11-14

**Authors:** Hengguang Shen, Zhaolin Zhu, Haotian Lu, Haonan Ju, Jinliang Huang, Zhihao Chen

**Affiliations:** 1 Hainan Institute of Zhejiang University, Sanya 572025, China; 2 Weihai Sunfull Geophysical Exploration Equipment Co., Ltd., Weihai 264209, China

**Keywords:** sandwiched piezoelectric accelerometer, seismic exploration, low-frequency, wide-band

## Abstract

A sandwiched piezoelectric accelerometer is developed and optimized for acquiring low-frequency, wide-band seismic data. The proposed accelerometer addresses the challenges of capturing seismic signals in the low-frequency range while maintaining a broad frequency response through the design of multi-stage charge amplifiers and a sandwiched structure. The device’s design, fabrication process, and performance evaluation are discussed in detail. Experimental results demonstrate its performance in amplitude and phase response characteristics.

## 1. Introduction

Seismic exploration plays a pivotal role in assessing subsurface geological structures. In onshore seismic acquisition, primary sensor options include moving-coil velocity geophones [1,2] and piezoelectric accelerometers [3,4]. Notably, the limited sensitivity and constrained dynamic range of moving-coil geophones have led to the preference for piezoelectric accelerometers in modern high-precision land seismic exploration.

In marine environments, both 4C ocean-bottom nodes [5,6] and dual-sensor transition-zone geophones [7] incorporate hydrophone-based pressure measurements. However, the distinct signal reception mechanisms of moving-coil geophones and hydrophones introduce signal misalignment issues, posing challenges for post-processing. In the context of 4C ocean-bottom nodes and dual-sensor geophones, choosing an accelerometer offers distinct advantages in resolving this inconsistency.

Piezoelectric film accelerometers [8,9], optical accelerometers [10], and MEMS [11,12,13] offer superior performance compared to piezoelectric crystal accelerometers. However, due to their intricate manufacturing processes and higher costs, mature products in the seismic exploration market are relatively scarce. Therefore, the primary focus here is on cost-effective piezoelectric crystal accelerometers.

Seismic piezoelectric accelerometers, which primarily rely on seismic mass block-induced thickness deformation to detect ground motion [4], face a persistent challenge: a constrained bandwidth for low-frequency signals. While accelerometers employing a cantilever beam and a sizable mass in bending mode offer advantageous low-frequency responsiveness and high sensitivity [14,15,16,17], they also maintain a restricted bandwidth.

In 2012, Levinzon designed an ultra-low-noise seismic piezoelectric accelerometer, utilizing a cylindrical double cantilever beam with a diameter of approximately 65 mm and a resonance frequency of around 370 Hz. However, while resonant accelerometers can mitigate nonlinearity at higher frequencies, they are less suitable for detecting subtle vibrations and can be complex [18,19]. Nevertheless, the pursuit of comprehensive insights into geological formations necessitates the integration of low-frequency, wide-band capabilities.

This paper presents the development of a simple sandwiched piezoelectric accelerometer designed to address this limitation. By incorporating a sensor sandwiched structure and a multi-stage charge amplifier circuit, this design aims to maintain excellent low-frequency response while improving the capacity to capture high-frequency seismic signals.

The paper is structured as follows. Firstly, the design of the multi-stage charge amplifiers is guided by an analysis of chip parameters to ensure precise parameter selection for both charge conversion and amplification circuits. Secondly, a simple sandwiched piezoelectric accelerometer is meticulously designed using a unique configuration of piezoelectric ceramics and a carefully crafted core structure within the accelerometer. Lastly, the design encompasses the integration of decoupling capacitors and zero-drift capacitors for optimal performance.

## 2. Methodology

The process of developing the low-frequency, wide-band land piezoelectric accelerometer underwent several critical phases with the objective of enhancing high-frequency capabilities while establishing a lower starting frequency. Seismic accelerometers typically feature integrated electronics and a core comprising piezoelectric sensors [19]. The proposed methodology includes the incorporation of a multi-stage charge amplifier circuit, the design of the sensor structure, and the implementation of testing procedures.

### 2.1. Charge Amplifier Circuit Implementation

The charge amplifier serves as a critical interface between the piezoelectric element’s output and the measurement system [20,21]. The circuit design focuses on minimizing noise, extending the dynamic range, and compensating for nonlinearities. Special attention was given to achieving a balanced trade-off between amplification and signal fidelity, enabling accurate signal representation across the targeted frequency range.

#### 2.1.1. Amplifier Circuit Theory

**Figure 1:**
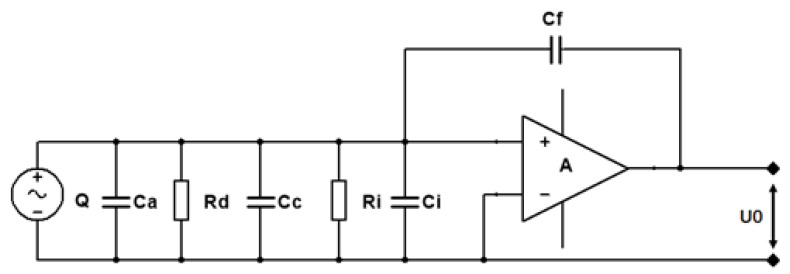
Equivalent circuit diagram of a sensor and a charge amplifier.

Figure 1 illustrates the equivalent circuit diagram that shows the connection between a sensor and a charge amplifier. It consists of the piezoelectric transducer and the charge amplifier used for signal amplification. The symbol A represents the open-loop gain of the operational amplifier, and Q represents the charge sensitivity from the signal source. Rd is the leakage resistance of the piezoelectric element, Ca is the capacitance of the sensor, Cc is the equivalent capacitance of the connecting cable, and Cf is the feedback capacitance of the charge amplifier. Additionally, Ri and Ci represent the input resistance and input capacitance of the amplifier, respectively, while Ui and Uo denote the input and output voltage of the amplifier. A charge amplifier is a specialized circuit designed to amplify charge signals. It typically features a very low bias current and high input impedance. In the design of the seismic piezoelectric accelerometer, Levinzon (2012) [19] utilized an ultra-low-noise FET-input charge amplifier.

In a charge amplifier, the output voltage is determined by the feedback capacitance and is directly proportional to the input charge quantity (charge sensitivity). This unique feature allows the charge amplifier to convert minuscule changes in charge into substantial voltage variations, thereby enhancing signal detectability and readability. Due to these operational characteristics, charge amplifiers are highly valuable in applications that require precision and low noise, such as precision measurements and sensor signal amplification. For the circuit shown in Figure 1, Rd and Ri can be neglected, and the output voltage of the amplifier is

(1) $$U_{o}=\frac{-A\cdot Q}{C_{a}+C_{c}+C_{i}}$$

When A>>1 and satisfies the condition $\left(1+A\right)C_{f}>10\left(C_{a}+C_{c}+C_{i}\right)$, then

(2) $$U_{o}=\frac{-Q}{C_{f}}$$

It can be observed that Uo is independent of $C_{a}$, $C_{c}$, and $C_{i}$. Moreover, when $C_{f}$ is determined, $U_{o}$ is only proportional to the output charge Q and is nearly unaffected by the length of the connecting cable.

#### 2.1.2. Multi-Stage Charge Amplifier and Operation

In practical applications, single-stage amplification circuits often struggle to meet the demands of real-world circuits. This is primarily because of considerations related to the operational amplifier’s amplification factor and the subsequent signal processing stages. To fulfill the required functionalities, there arises a necessity to design multi-stage amplifiers by combining several single-stage amplifiers.

**Figure 2:**
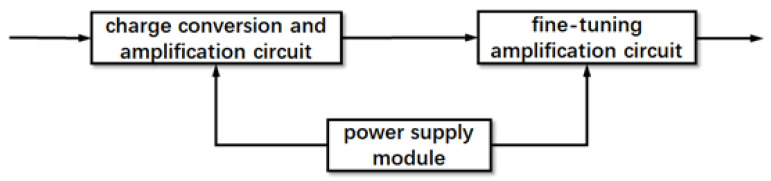
Multi-stage charge amplifier modules.

The designed charge amplifier circuit structure, as illustrated in Figure 2, comprises three key components: the charge conversion and amplification circuit, the fine-tuning amplification circuit, and the power supply module. Initially, the high-impedance signal output from the piezoelectric sensor is transformed into a low-impedance signal. However, due to parameter variations among piezoelectric crystals, a fine-tuning amplification circuit is integrated to ensure consistency in the final output parameters. This circuit performs a secondary normalization process on the amplified signal. Simultaneously, the power supply module provides a direct current power source to the electronic components and amplifier chips within the circuit.

**Figure 3:**
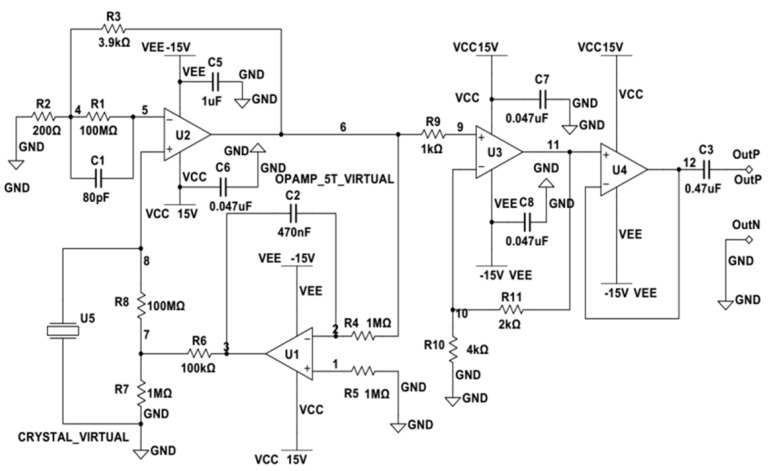
Multi-stage charge amplifier circuit.

In functionality, the amplification circuit, illustrated in Figure 3, is primarily comprised of the LT1169 and LM2904 chips. The LT1169, a dual-channel, JFET(Junction Field-Effect Transistor)-input precision operational amplifier manufactured by Linear Technology (now part of Analog Devices), stands out for its low noise and low bias current characteristics. As for the power supply module, it relies on a DC/DC module with the model number A1215XT-1WR2.

As illustrated in Figure 3, the delicate charge signal generated by the piezoelectric crystal U5 is directly fed to the input of operational amplifier U2 via connecting leads. The fundamental same-phase proportional amplification circuit is established by resistors R2 and R3 in conjunction with operational amplifier U2. This configuration includes negative feedback via resistor R3, forming a negative feedback amplification circuit. In parallel, the combination of capacitor C2, resistor R4, and operational amplifier U1 constitutes a conventional integrator circuit that transforms charge variations into voltage signals. As a result, this circuit not only accomplishes the conversion of charge to voltage signals, but also initiates the initial amplification of weak signals. The output of U2 is linked to the input of U3 through resistor R9, creating a fundamental same-phase proportional amplification circuit together with resistors R10 and R11 in tandem with operational amplifier U3. The feedback loop is accomplished via resistor R11, resulting in a negative feedback amplification circuit. The signal is subsequently directly routed to the inverting input of operational amplifier U4, which inherently acts as a voltage follower circuit, thereby further enhancing voltage signal amplification and processing within the circuit.

Within the circuit, high-frequency decoupling capacitors are affixed near 4 pins VSS and 8 pins VCC of the LT1169 and LM2904 chips. These capacitors serve to mitigate the impact of the power supply on the operational amplifiers. They effectively filter out high-frequency disturbances, heighten anti-interference capabilities, and guarantee stable amplifier operation by averting oscillations. The strategic placement of these capacitors in close proximity to the chip’s power supply terminals aids in eradicating rectification noise from the analog circuit. For this purpose, ceramic capacitors within the 0.01 μF to 0.1 μF range are suitable, with an actual selection of a 0.047 μF ceramic capacitor for the practical circuit. Capacitor C3 is judiciously included in the circuit to isolate zero drift when the sensor output is active. In the integrator circuit, tantalum capacitors are preferred for capacitor C2 due to their extended lifespan and high-temperature resistance attributes.

In the design of a multi-stage charge amplifier, we carefully considered the advantages and cost factors associated with JFET devices. Ultimately, we selected two dual-channel chips, LT1169 and LM2904, to fulfill the functions of charge conversion and signal amplification. Furthermore, to ensure the stable operation of the circuit board and enhance its resistance to interference, we implemented a modular power supply connection and protective design. Additionally, we optimized the design of decoupling capacitors and zero-drift capacitors.

### 2.2. Design of the Sensor Sandwiched Structure

The cornerstone of the accelerometer’s enhanced performance lies in the innovative sensor sandwiched structure. This design introduces a well-thought-out arrangement of materials and components to optimize both low-frequency sensitivity and high-frequency response. The sensor sandwiched structure not only supports the mechanical properties of the cantilever beam but also minimizes the effects of nonlinearity. Comprehensive engineering and design considerations were undertaken to ensure the effective coupling of the piezoelectric material with the seismic mass block, thereby enhancing signal transmission and reducing undesirable artifacts.

#### 2.2.1. Piezoelectric Element Selection

Piezoelectric ceramics, especially those based on zirconate titanate (PZT) [22,23], are well-regarded materials in the field of piezoelectricity. When compared to quartz crystals, piezoelectric ceramics offer significantly higher piezoelectric coefficients. They are characterized by advantages such as ease of fabrication, resistance to moisture and high temperatures, and ease of shaping. Moreover, they come at a low manufacturing cost. Piezoelectric ceramics find numerous applications across various industrial sectors.

In the design of the sandwiched piezoelectric accelerometer discussed in this paper, PZT ceramics were chosen as the ideal material for the piezoelectric elements. This selection is grounded in their exceptional piezoelectric properties, ease of manufacturing, robustness in various environmental conditions, and cost-effectiveness. These characteristics collectively make PZT ceramics a fitting choice to ensure the accelerometer’s optimal performance and suitability for seismic signal detection in a wide range of applications.

#### 2.2.2. Piezoelectric Ceramic Assembly

**Figure 4:**
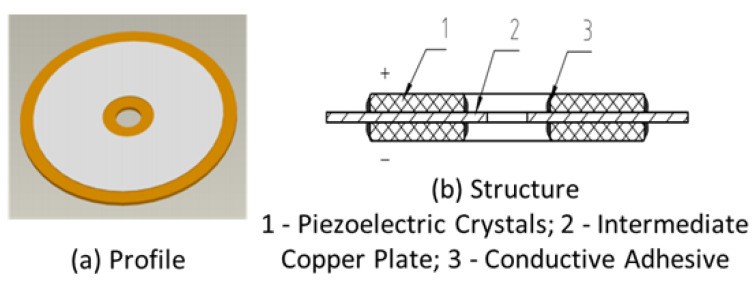
Piezoelectric ceramic assembly.

In Figure 4a, the accelerometer’s core contains a PZT-5 piezoelectric crystal with a fundamental resonance frequency of approximately 2 kHz. In this paper, the ideal specifications for the piezoelectric crystal have been determined: a thickness of 0.2 mm, an outer diameter of 33.3 mm, and an inner diameter of 9 mm. For the intermediate plate, H59 brass is selected, boasting a thickness of 0.7 mm and a diameter of 38 mm. The central through-hole in the intermediate plate measures 4.5 mm in diameter.

The innovative piezoelectric ceramic assembly centers around an intermediate plate housing two piezoelectric crystals in a sandwich structure in Figure 4b. These identical circular crystals, differing solely in polarity, are appropriately connected in series using conductive adhesive on a copper plate. An adhesive ring forms at the intersection of the exposed surfaces of the piezoelectric crystal’s electrode angles, effectively mitigating potential disturbances from reflections of inward-bound spurious signals. Both sides of the piezoelectric crystal’s exposed surfaces undergo a silver-plating process. Precisely, the positive electrode lead is soldered to the positive pole of the exposed piezoelectric crystal, while the negative electrode lead is similarly connected to the negative pole of the exposed crystal.

#### 2.2.3. Sandwiched Core of the Accelerometer

**Figure 5:**
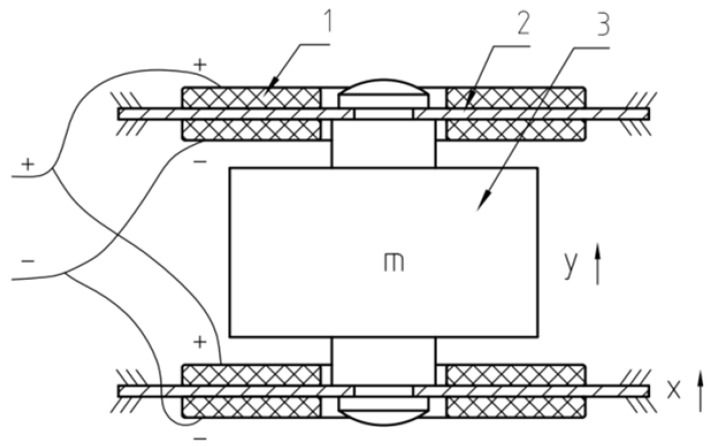
The core of a sandwiched piezoelectric accelerometer; 1—piezoelectric crystal; 2—intermediate copper plate; 3—mass.

The design of the sandwiched piezoelectric accelerometer core seeks a balance between generating significant charge output from the piezoelectric crystals and maintaining substantial overall capacitance. The approach involves a stepwise process: initially, two piezoelectric crystals are connected in series with an intermediate plate. Later, two identical novel piezoelectric ceramic assemblies are connected in parallel to form the core, as shown in Figure 5.

The central part of the design features an inertial mass shaped like a truncated cone (“m”). This mass, made from high-density brass weighing 34.2 g, is rigidly attached to the piezoelectric ceramic assemblies using screws. This setup ensures that the polarities of the ceramic crystals align and restrict any motion of the intermediate plate.

The chosen configuration achieves enhanced sensitivity by connecting four piezoelectric crystals first in series and then in parallel, doubling the output charge and voltage. When the ground and housing vibrate, the inertia of the mass causes minor displacement relative to the housing. This displacement induces parallel deformations in the ceramic assemblies, generating output charges at the lead ends.

The design achieves both enhanced electrical performance and stable mechanical vibration. The careful selection of materials and connection methods ensures robust charge and voltage output, while still being compatible with existing hydrophone setups for mixed data collection scenarios. The compactness of the product is also prioritized.

In summary, the optimized design of the sandwiched piezoelectric accelerometer core combines series and parallel connections of piezoelectric components with an inertial mass. This achieves heightened sensitivity, robust charge and voltage output, and compatibility with existing data acquisition setups while maintaining a compact form factor.

### 2.3. Response Analysis

**Figure 6:**
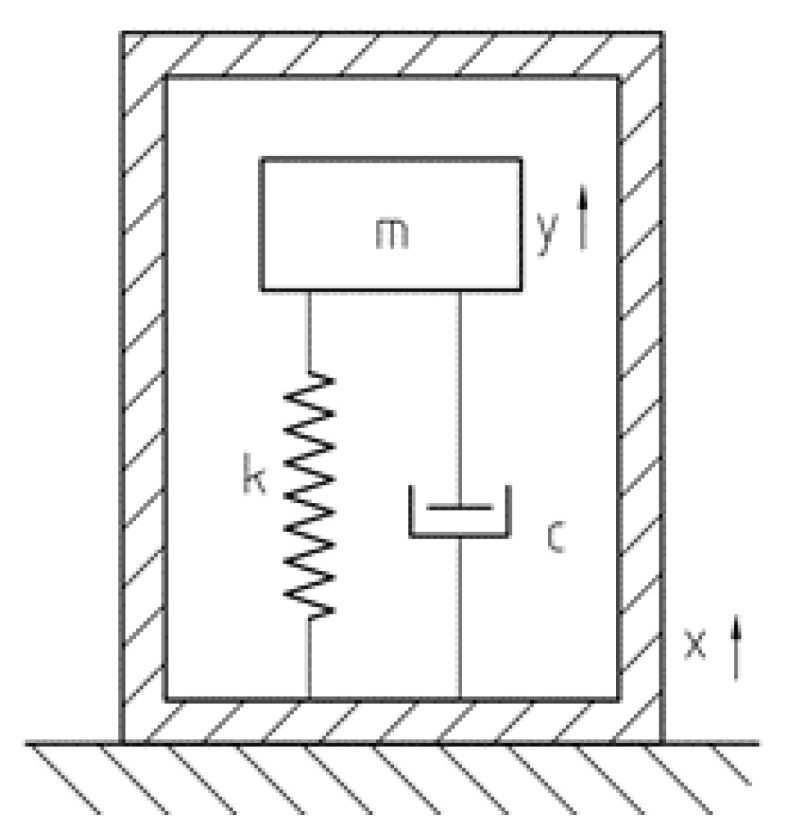
Accelerometer dynamics model.

All the types of the accelerometers can be approximately described with the one degree of freedom model [14]. As illustrated in Figure 6, the model includes the mass block m, the spring k, and the damping c. The variable x represents the housing displacement, and they represents the mass displacement to the housing.

The motion equations for a second-order mechanical system of the above model (Figure 6) can be written as

(3) $$\frac{\partial^{2}y}{\partial t^{2}}+2\varsigma \omega_{n}\frac{\partial y}{\partial t}+\omega_{n}^{2}y=-\frac{\partial^{2}x}{\partial t^{2}}$$

where, ω is the angular frequency, $\omega_{n}$ is the natural frequency, and ς is the damping ratio. By performing Fourier transforms on both sides of the above equation, we can obtain the following equation:

(4) $$-\omega^{2}+2j\zeta \omega \omega_{n}+\omega_{n}^{2}=\omega^{2}\frac{x}{y}$$

So, the response characteristics of an accelerometer can be derived as follows:

(5) $$H\left(\omega \right)=\frac{y(\omega )}{x(\omega )}=\frac{{\left(\frac{\omega }{\omega_{n}}\right)}^{2}}{1-{\left(\frac{\omega }{\omega_{n}}\right)}^{2}+2j\varsigma \left(\frac{\omega }{\omega_{n}}\right)}$$

where, y(ω) represents the Fourier Transform of the output of the inertial mass relative to the housing (output). x(ω) represents the Fourier Transform of the housing’s motion input. We can further obtain the amplitude and phase characteristics:

(6) $$\left|H\left(\omega \right)\right|={\left(\frac{\omega }{\omega_{n}}\right)}^{2}\frac{1}{\sqrt{{\left[1-{\left(\frac{\omega }{\omega_{n}}\right)}^{2}\right]}^{2}+4\varsigma^{2}{\left(\frac{\omega }{\omega_{n}}\right)}^{2}}}$$

and

(7) $$\varphi \left(\omega \right)=-arctan\frac{2\varsigma \left(\frac{\omega }{\omega_{n}}\right)}{1-{\left(\frac{\omega }{\omega_{n}}\right)}^{2}}$$

Considering the high natural frequency of piezoelectric ceramic crystals, typically falling within the range of 500 to 3000 Hz, it is noteworthy that the uppermost frequencies encountered in seismic exploration—usually associated with intermediate and deep layers—are considerably lower than this frequency range. Additionally, the absence of coil structures in the sandwiched piezoelectric accelerometer component eliminates the occurrence of eddy current damping. By opting for high-performance piezoelectric crystals or employing judicious combinations through series and parallel connections, an effectively elevated damping value is achieved.

**Figure 7:**
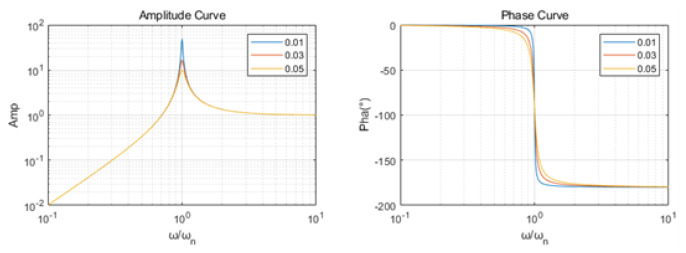
Response characteristics of the accelerometer with damping ratios of 0.01, 0.03, and 0.05.

By using Formulas (6) and (7), Figure 7 shows the response characteristics of the accelerometer: (a) amplitude–frequency curve; (b) phase–frequency curve. From the amplitude–frequency and phase–frequency characteristics of accelerometers, the practical effective frequency bandwidth for seismic exploration (ranging from 1 to 300 Hz) exhibits a linear amplitude–frequency curve and an equally linear phase–frequency curve. This implies that the sandwiched piezoelectric accelerometer theoretically possesses the capability to provide a proportionate output response according to the input signal’s frequency, while maintaining a consistent phase alignment, essentially remaining at a constant value irrespective of changes in the $\omega /\omega_{n}$ ratio.

## 3. Fabrication and Analysis

### 3.1. Accelerometer Fabrication

**Figure 8:**
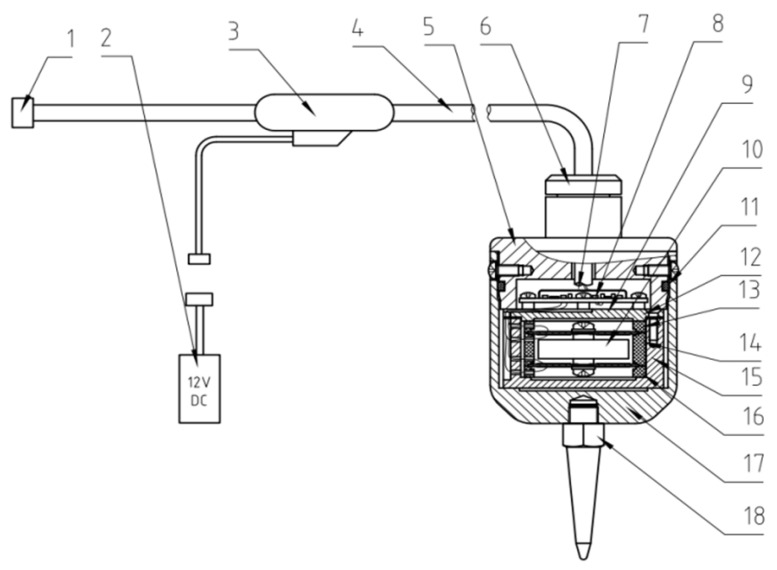
Structural components of the sandwiched piezoelectric accelerometer: 1—connector; 2—power supply; 3—header; 4—cable; 5—upper cover; 6—plug; 7—shielding cover; 8—circuit board; 9—upper pressure block; 10—accelerometer core; 11—sealing ring; 12—upper pressure plate; 13—upper clamping body; 14—middle clamping body; 15—inner core shell; 16—lower clamping body; 17—housing; 18—tail cone.

The complete configuration of the sandwiched piezoelectric accelerometer encompasses distinct components: the accelerometer core, charge amplifier, housing, tail cone, cables, etc., as showed in Figure 8.

The accelerometer’s core incorporates a novel arrangement of piezoelectric ceramic assemblies, strategically connected in a series-parallel configuration as illustrated in Figure 5. This design choice aims to optimize charge and voltage output. These innovative ceramic assemblies are precisely positioned within the limit slots of the upper, middle, and lower clamping bodies made of polysulfone material. Sequential compression and stabilization are achieved by the metal upper pressure plate and the inner core shell, effectively anchoring the clamping bodies. This meticulous arrangement ensures the stability of the entire structure during resonance motion, preventing any undesirable rotation or vibration. The use of non-metallic upper, middle, and lower clamping bodies, along with their spacing and limiting attributes, effectively curbs any undesired rotation or deviation of the novel piezoelectric ceramic assemblies, thus maintaining their consistent operational state.

The finely tuned charge amplifier circuit board is securely fastened onto the upper pressure plate of the accelerometer’s core using screws. On the upper surface of the circuit board, a metal shielding cover is skillfully soldered. This strategic arrangement isolates the metal upper pressure plate from the metal shielding cover, effectively mitigating electromagnetic interference from the surrounding environment on the charge amplifier. The secure fastening of the circuit board ensures that components within the protective shell system, even during resonance motion, do not produce any noise that could disrupt the core’s intended resonant behavior. Additionally, the metal shielding plate on the circuit board plays a crucial role in safeguarding the circuit against potential magnetic field interference, ensuring the utmost fidelity in the conversion and amplification circuits.

**Figure 9:**
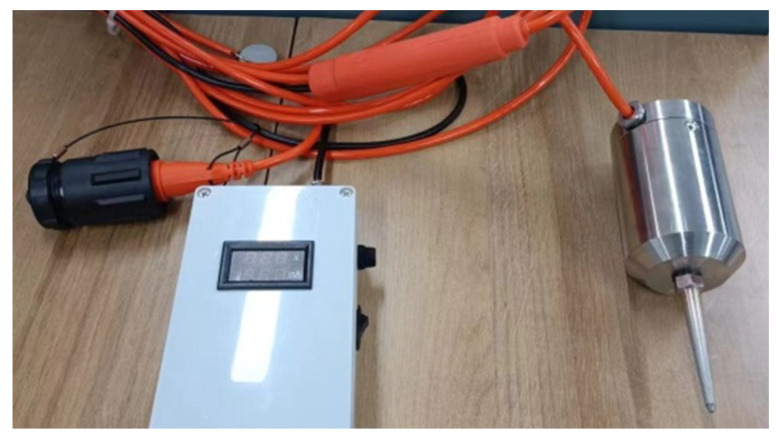
The prototype of the sandwiched piezoelectric accelerometer.

The external housing, upper cover, and sealing ring form a robust outer layer, offering comprehensive protection to the core against compression and ensuring effective insulation. Within the transmission cable, one pathway is dedicated to the power line. Through the header, this line connects to an external 12 V DC power source, supplying power to the internal charge amplifier circuit board. Simultaneously, another pathway accommodates the signal line, linking through the connector to the instrument. This facilitates the smooth transmission of amplified signals from the charge amplifier. Recognizing that the accelerometer primarily engages in information collection once deployed, the external power source is typically not directly connected to the power terminal of the header. Instead, it can be centralized for charging or storage, only establishing a connection to the relevant plug when the accelerometer is actively required to power the circuit board. The accelerometer prototype is as shown in Figure 9.

### 3.2. Sandwiched Accelerometer Characteristic Fitting Curve

Using the conventional moving-coil velocity geophone SM-24 as our reference, which has a single-unit sensitivity of 20.9 V/m/s, we observe that the modified configuration, SM-24 with five in series and two in parallel (SM-24-5S2P), exhibits a sensitivity of 104.5 V/m/s.

To evaluate a sensor’s velocity sensitivity, we employ a vibration table. This apparatus imparts controlled velocity vibrations, with the sensor connected to it. By measuring the signal output voltage from the sensor, we can precisely determine its sensitivity and its response to varying vibration velocities.

Additionally, the vibration table proves invaluable in assessing the sensor’s acceleration sensitivity. It generates controlled accelerations, and the sensor remains connected. Measuring the signal output voltage from the sensor allows us to deduce the sensor’s sensitivity and its response to fluctuating vibration accelerations.

**Figure 10:**
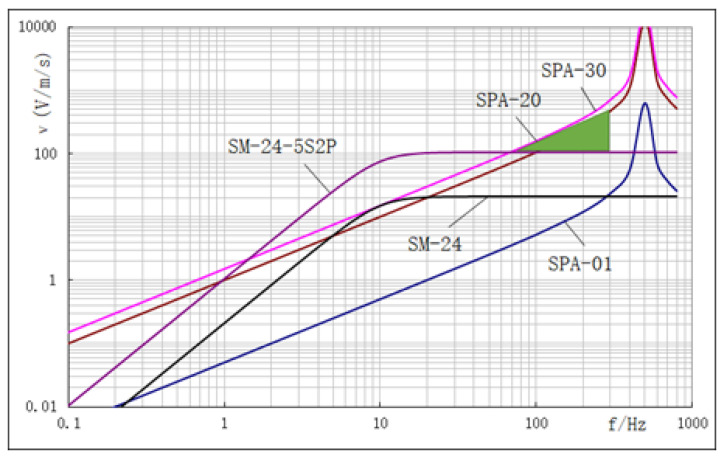
Comparison of amplitude–frequency characteristics among different SM-24 geophones and accelerometers.

In this study, our primary focus is on monitoring velocity sensitivity for a comparison between SM-24 and the sandwiched piezoelectric accelerometer. To achieve a better and matched output voltage with SM-24, we utilize different charge amplifier gains, labeled SPA-01, SPA-20, and SPA-30, each with gains of 1, 20, and 30 times, respectively, for the accelerometers. Through practical vibration table sensitivity measurements at different frequencies across different sensors and the creation of smooth, approximate relationship curves via data fitting and extension, based on the theoretical amplitude–frequency response of the moving-coil velocity geophone and the sandwiched piezoelectric accelerometer, we can present an approximate comparison curve illustrating their amplitude–frequency, as depicted Figure 10.

Here, there is a SM-24 natural frequency of 10 Hz and a damping coefficient of 0.686. The one and the curves resulting from SM-24-5S2P consistently exhibit an inflection point at the 10 Hz frequency. Their curves’ initial portion follows a diagonal pattern, while the latter part adopts a horizontal trajectory. Furthermore, given the common spurious frequency point around 200 Hz, within the effective seismic exploration frequency range of 1 Hz to 300 Hz, the suitable operational span is limited to 10 Hz to 200 Hz. In Figure 10, when comparing with SM-24, the amplitude–frequency characteristic curve of the sandwiched piezoelectric accelerometer SPA-01 is nearly a slanted straight line within a limited frequency range in seismic exploration. Additionally, for weak charge release by the piezoelectric ceramic, the characteristic curve exhibits lower voltage values when not connected to the charge amplifier (specifically, SPA-01 with the charge amplifier gain set to 1). Thus, SPA-01 falls short of achieving the sensitivity displayed by a single SM-24 accelerometer. However, a significant enhancement occurs for SPA-20, that is to say, the charge amplifier gain reaches 20 times, resulting in a notable shift of the characteristic curve. While SPA-20 still trails behind the performance of a single SM-24 accelerometer around the natural frequency point of 10 Hz, the SPA-30 characteristic curve attains superiority as the amplifier gain is further elevated to 30 times, particularly after incorporating a finely tuned amplification circuit. Additionally, as indicated by the green shaded region in the graph, the performance of SPA-30 can even surpass the effectiveness of the SM-24-5S2P within the seismic exploration frequency range spanning from 50 Hz to 300 Hz.

During seismic exploration, the filtering and attenuation effects of the Earth’s surface result in a predominance of low-frequency signals, with high frequencies missing and submerged in noise. As the sensing end of seismic exploration, sensors should strive to retain as many high-frequency signals as possible. This effectively broadens the usable seismic frequency band and enhances the detector’s dynamic range. As illustrated in Figure 10, when contrasting the moving-coil geophone, the amplitude–frequency characteristic curve of the designed accelerometer, following the natural frequency 10Hz, realizes the amplification of weak signals and undergoes significant enhancement across a wide frequency range. Simultaneously, the accelerometer curve, exhibiting a diagonal relationship with frequency, manifests greater amplification for high-frequency signals in comparison to low-frequency signals. This phenomenon mitigates the scarcity of high-frequency data in contrast to low-frequency data in seismic exploration, effectively suppressing low-frequency signals while elevating the prominence of high-frequency signals.

### 3.3. Frequency Sweep Testing

According to relevant standards, the sensitivity of geophones is typically tested at a vibration frequency of 80Hz and a velocity of 0.7 in/s (approximately 1.8 cm/s) using a vibration table. In this experiment, the DYS-200-2 electric vibration system produced by Suzhou Test Instrument Factory was employed to conduct frequency sweep testing on the SM-24-5S2P, the sandwiched piezoelectric accelerometers SPA-20 and SPA-30.

**Figure 11:**
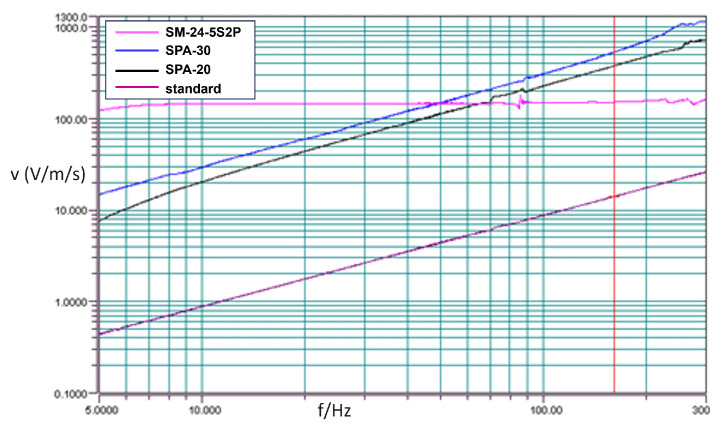
Frequency sweep curves of multiple accelerometers.

**Figure 12:**
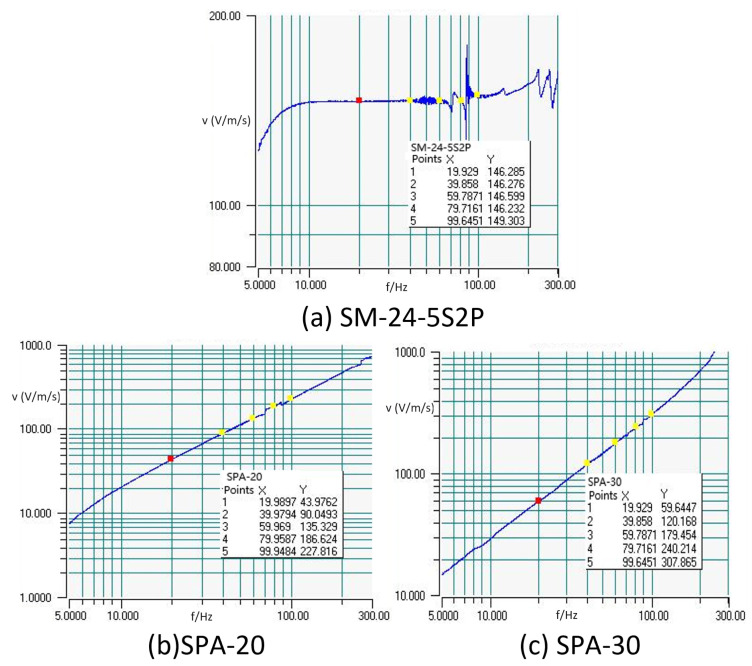
Frequency point values.

Based on the actual testing conditions of the vibration table, the testing velocity was set to 1.4 cm/s. As shown in Figure 11, the pink line is SM-24-5S2P, blue line is SPA-30 with 279.7 g, the black line is SPA-20 with 85.9 g, and the purple line is the standard sensor of the vibration table. Within the frequency range of 10 Hz to 300 Hz, the frequency sweep curve for the SM-24-5S2P forms a horizontal line under constant velocity scanning, while the curve for the piezoelectric accelerometers exhibits a consistently rising diagonal line, with similar slopes throughout the frequency range. The vibration table can output the correct value of different frequency points for the curves in Figure 11, which are showed in the following figures:

In Figure 12, it is evident that for SM-24-5S2P, the approximate output values are 146.276 at 40 Hz and 146.232 at 80 Hz. These values are remarkably close, demonstrating a consistent pattern. The sensitivity corresponding to the current frequency is calculated as 104.43 V/m/s using the formula: 146.232 × 10 mV/1.4 cm/s. At 80 Hz, SPA-20 records a measured output value of 186.624, resulting in a sensitivity of 133.3 V/m/s, also calculated using a similar formula. As for SPA-30, the measured output value at 80 Hz is 240.214, leading to a sensitivity of 171.58 V/m/s, again calculated using a similar formula.

Based on the calculations above, the octave bandwidth of the sandwiched piezoelectric accelerometer is approximately 6 dB, and its sensitivity during the frequency sweep is higher than that of the SM-24-5S2P at 80 Hz. This verifies that by designing a well-structured charge conversion and amplification circuit, coupled with fine-tuning amplification, it is possible to normalize the accelerometer cores into consistently well-matched end products.

**Figure 13:**
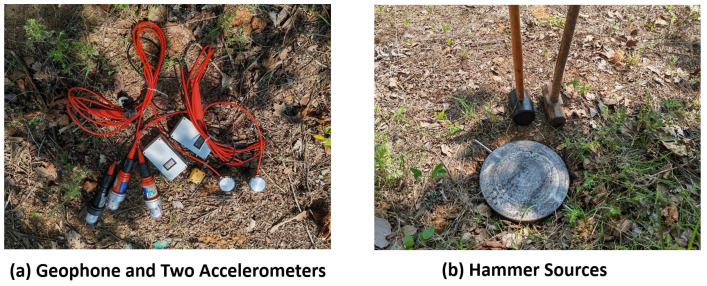
Field data acquisition from hammer sources.

**Figure 14:**
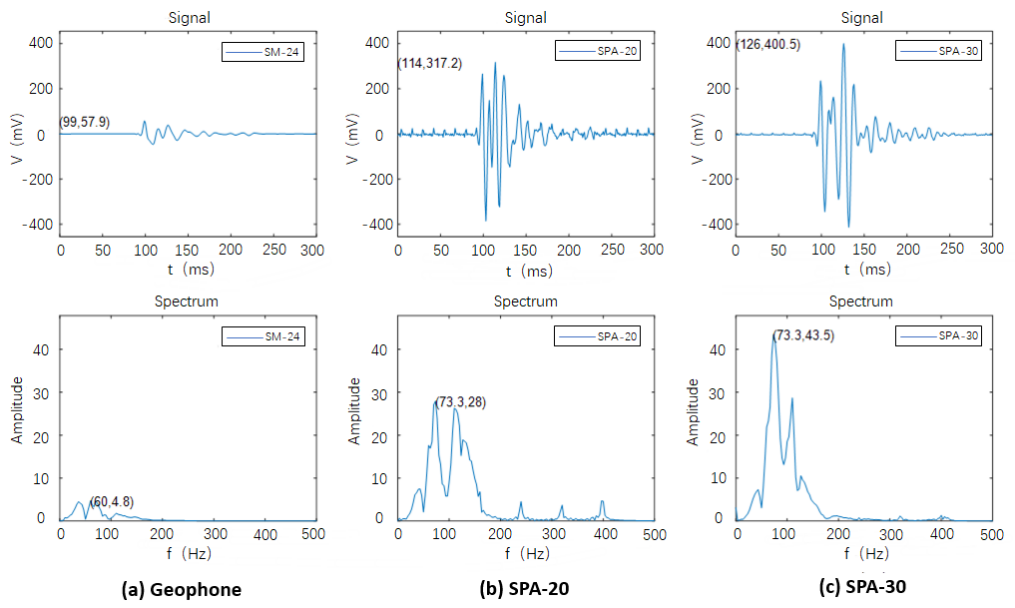
Acquired signals from geophone and two accelerometers.

Figure 13 illustrates the configuration of the geophone combination, along with two designed accelerometers (SPA-20 and SPA-30), the hammer sources. Analyzing the acquired signals in Figure 14, it becomes evident that the specially designed sandwiched piezoelectric accelerometer featured here excels in capturing signals with significantly larger amplitudes, heightened sensitivity, increased high-frequency content, and a broader frequency range compared to traditional moving-coil detectors. Unlike the fixed amplification factor inherent to moving-coil detectors, the piezoelectric detector’s amplification factor can vary, influenced by factors such as signal strength and frequency. Typically, this amplification factor falls within the range of four to six times that of traditional detectors. Referring to Figure 14b,c, when the piezoelectric detector is activated, it introduces approximately 110 Hz or 90 Hz harmonic interference, which adversely impacts signal quality. Subsequent steps will be implemented to effectively shield this interference signal.

## 4. Discussion

In the pursuit of precision during the processes involving piezoelectric crystal cutting, bonding, silver plating, and aging, achieving consistent results within a given batch has proven to be a complex challenge. This complexity has led to notable variations and scatter in the distribution of parameters. To address this issue effectively, additional efforts should be directed towards the development of specialized tooling and fixtures designed for the convenient testing and classification of the novel piezoelectric ceramic assemblies.

This strategic approach will enable us to pair these assemblies with different inertial mass blocks, ensuring the stability and uniformity of critical parameters. Such steps are crucial for enhancing the overall performance and reliability of our technology. Moreover, to meet the demands of digital applications and further explore the extension of low-frequency and bandwidth capabilities, the incorporation of analog-to-digital conversion and feedback circuits is also imperative.

The influence of the ionosphere on seismic sensors [24], particularly for low-frequency operation, is a topic recommended by some experts and is of great interest in seismic acquisition.

Continued research and development in these areas promise to refine our processes, ensuring consistent and high-quality outcomes, and expanding the potential applications of our technology.

## 5. Conclusions

By fitting characteristic curves and comparing them, the contrast and trend in sensitivity between the sandwiched piezoelectric accelerometer and the velocity geophone become much clearer and comprehensible. Despite sharing the same motion differential equation, these two accelerometers differ significantly in their elastic element stiffness (K), resulting in natural frequencies that are tens to hundreds of times apart. Nevertheless, both of them exhibit excellent linearity in frequency ranges well beyond their respective natural frequencies.

Building upon this understanding, a concept was proposed to expand the frequency bandwidth of seismic detectors by enhancing the detection of high-frequency signals. This concept harnessed the acceleration characteristics of piezoelectric materials to achieve our objectives, culminating in the development of the sandwiched piezoelectric accelerometer for land-based applications. Through comparative analysis, it demonstrated significant capabilities in suppressing low-frequency signals and improving the detection of high-frequency signals.

Furthermore, notable improvements were made to a charge amplifier circuit, which is primarily divided into a charge conversion amplification circuit and a fine-tuning amplification circuit. During the design process, a thorough comparison of the technical parameters of operational amplifier chips was conducted to select suitable chips for fulfilling the intended functions. Additionally, decoupling capacitors and zero-drift capacitors were integrated into the circuit design to enhance its overall performance.

To further validate the testing results and confirm the fulfillment of initial design objectives, a frequency sweep test was conducted on the vibration table. In field hammer source testing, in contrast to the velocity geophone, the sandwiched piezoelectric accelerometer lacks natural frequency points within the effective seismic frequency range. The outcomes of the vibration table’s frequency sweep test underscore how the latter’s 6 dB octave bandwidth rise rate effectively attenuates low frequencies and accentuates high frequencies, thus significantly enhancing its overall performance.

## Data Availability

The data that support the findings of this study are available upon reasonable request from the corresponding author.
